# Reduced T2*-weighted placental MRI predicts foetal growth restriction in women with chronic rheumatic disease—a Danish explorative study

**DOI:** 10.1007/s10067-024-06889-5

**Published:** 2024-04-26

**Authors:** Thea Vestergaard, Mette Julsgaard, Rikke Bek Helmig, Emilie Faunø, Tau Vendelboe, Jens Kelsen, Trine Bay Laurberg, Anne Sørensen, Bodil Ginnerup Pedersen

**Affiliations:** 1 Department of Hepatology and Gastroenterology, Aarhus University Hospital, Palle Juul-Jensens, Boulevard 99, Entrance C, Level 1, Fix-Point C117, 8200 Aarhus, Denmark; 2 Institute of Clinical Medicine, Aarhus University, Aarhus, Denmark; 3 Center for Molecular Prediction of Inflammatory Bowel Disease (PREDICT), Department of Clinical Medicine, Aalborg University, Aalborg, Copenhagen Denmark; 4 Department of Obstetrics and Gynaecology, Aarhus University Hospital, Aarhus, Denmark; 5 Department of Radiology, Aarhus University Hospital, Aarhus, Denmark; 6 Department of Rheumatology, Aarhus University Hospital, Aarhus, Denmark; 7 Department of Obstetrics and Gynaecology, Aalborg University Hospital, Aalborg, Denmark

**Keywords:** Foetal growth restriction, MRI, Placental dysfunction, Pregnancy, Rheumatology

## Abstract

**Objectives:**

Women with chronic rheumatic disease (CRD) are at greater risk of foetal growth restriction than their healthy peers. T2*-weighted magnetic resonance imaging of placenta (T2*P-MRI) is superior to conventional ultrasonography in predicting birth weight and works as a proxy metabolic mirror of the placental function. We aimed to compare T2*P-MRI in pregnant women with CRD and healthy controls. In addition, we aimed to investigate the correlation between T2*P-MRI and birth weight.

**Methods:**

Using a General Electric (GE) 1.5 Tesla, we consecutively performed T2*-weighted placental MRI in 10 women with CRD and 18 healthy controls at gestational week (GW)24 and GW32. We prospectively collected clinical parameters during pregnancy including birth outcome and placental weight.

**Results:**

Women with CRD had significantly lower T2*P-MRI values at GW24 than healthy controls (median T2*(IQR) 92.1 ms (81.6; 122.4) versus 118.6 ms (105.1; 129.1), *p* = 0.03). T2*P-MRI values at GW24 showed a significant correlation with birth weight, as the T2*P-MRI value was reduced in all four pregnancies complicated by SGA at birth. Three out of four pregnancies complicated by SGA at birth remained undetected by routine antenatal ultrasound.

**Conclusion:**

This study demonstrates reduced T2*P-MRI values and a high proportion of SGA at birth in CRD pregnancies compared to controls, suggesting an increased risk of placental dysfunction in CRD pregnancies. T2*P-MRI may have the potential to focus clinical vigilance by identifying pregnancies at risk of SGA as early as GW24.

**Table:** 

**Key Points** • *Placenta-related causes of foetal growth restriction in women with rheumatic disease remain to be investigated*. • T2*P-MRI values at gestational week 24 predicted foetuses small for gestational age at birth. • T2*P-MRI may indicate pregnant women with chronic rheumatic disease (CRD) in need of treatment optimization.

**Supplementary Information:**

The online version contains supplementary material available at 10.1007/s10067-024-06889-5.

## Introduction

The clinical disease course of chronic rheumatic diseases (CRD) such as rheumatoid arthritis (RA) and psoriatic arthritis (PsA) is often favourable during pregnancy, yet for ankylosing spondylitis (AS) to a lesser extent [1–3]. However, the systemic inflammatory load of the diseases may persist in quiescence and influence the gestation period, possibly affecting the growth of the foetus. Women who experience disease activity during pregnancy are at even higher risk of preterm birth, foetal growth restriction and giving birth to children of lower birth weight than women experiencing remission throughout pregnancy [2–5]. Of note, irrespective of disease activity, women with RA give birth to children with lower birth weight than the background population [6]. Lower birth weight (below 3400 g) is associated with adverse outcomes such as diabetes and cardiovascular disease later in life, and it therefore remains of great interest to uncover the reason for this discrepancy [7, 8]. However, the origin of the growth restriction in children of women with RA remains undisclosed, and it is conceivable that dysfunction of the placenta is involved, as this in several studies has been shown to be the case in healthy women bearing children displaying intrauterine growth restriction [9, 10].

Clinical assessment of placental function remains challenging. The ultrasonography assessments performed in the antenatal care of pregnant women provide indirect measurements of the function of the placenta by estimating blood flow in the umbilical and uterine arteries and foetal growth [11]. Ultrasonography is cheap and enjoys wide acceptance by pregnant women, although ultrasonography assessment of women at risk of foetal growth restriction does usually not allow firm conclusions until the 3rd trimester when the foetal growth is manifestly reduced, thereby minimising the window of therapeutic optimization.

The placental MRI transverse relaxation time, T2*, is an established non-invasive in vivo imaging of placental function. Moreover, studies indicate that placental T2* MRI may outperform the otherwise accepted gold standard ultrasonography in the prediction of low birth weight [12]. T2*-weighted imaging of the placenta reflects tissue oxygenation as the presence of deoxyhaemoglobin affects the spins of protons thereby creating magnetic field inhomogeneity, which will reduce the T2* value. In the hypoxic tissue of a dysfunctional placenta, the T2* value is reduced. However, T2* values are dynamic and decrease physiologically as the healthy pregnancy progresses [13, 14]. Placental T2* MRI is closely associated with clinical outcomes like pre-eclampsia and being small for gestational age (SGA) [9]. In that sense, MRI may both serve as imaging and a proxy-metabolic mirror of the placenta, and several studies have shown the birth weight of the foetus to be positively associated with the T2* value [10].

Data on T2*-weighted MRI of the placenta in women with CRD do not exist, and the aim of the present study was to compare consecutive T2*-weighted MRI scans at W24 or W32 of women with CRD and healthy controls. The secondary aim was to assess a potential correlation between T2* values and clinical outcomes.

## Methods

The study was approved by the Danish Data Protection Agency (j.nr: 1–16-02–370-18) and the Ethics Committee of the Central Region of Denmark (j.nr: 1–10-72–289-18). All patients provided written informed consent.

All pregnant women were prospectively enrolled in the study before 12 weeks of gestation at a tertiary hospital between 2019 and 2021.

The women with CRD were enrolled from the outpatient clinic of the Department of Rheumatology, Aarhus University Hospital, Denmark. Healthy controls were enrolled at the Department of Obstetrics and Gynaecology, Aarhus University Hospital, Denmark.

Participants in the respective groups were matched in age, BMI, parity and smoking status, and we aimed at a 1:2 ratio of CRD:healthy participants.

Inclusion criteria included ICD-10 assigned diagnosis of chronic rheumatic disease in the form of rheumatoid arthritis, psoriatic arthritis or ankylosing spondylitis; both pregnancy and rheumatic disease followed at the departments of rheumatology and obstetrics at Aarhus University Hospital, respectively, and age > 18. The only exclusion criterium was the previous history of pregnancies affected by growth retardation.

For each participant, we registered parity, smoking during pregnancy (yes/no), maternal weight before pregnancy, height, any prevalent comorbidities and whether the woman developed gestational diabetes or pre-eclampsia during pregnancy. We also registered any ongoing medications during pregnancy. For the women with CRD, we registered any rheumatic disease activity in any of the three trimesters. When assessing the presence of disease activity, we employed the Physician’s Global Assessment (PGA) to define dichotomously whether or not a woman experienced disease activity. The PGA is an arbitrarily designed, multicomponent measure of disease activity that is often used in prospective studies [15–17]. The PGA was based on the attending rheumatologist’s assessment incorporating anamnesis, clinical examination, escalation in medical treatment, blood test, e.g. C-reactive protein (CRP) and patient completion of validated disease activity indices: Physician’s Global Assessment of disease activity scale 0–100, Health Assessment Questionnaire, Disease Activity Score in 28 joints, Clinical Disease Activity Index, Bath Ankylosing Spondylitis Disease Activity Index, Bath Ankylosing Spondylitis Functional Index, Bath Ankylosing Spondylitis Metrology Index and Ankylosing Spondylitis Disease Activity Score [18–22]. To maintain consistency, the PGA was done by a single person blinded for EFW, obstetric outcomes and placental data, and assessments were performed during each trimester of pregnancy.

At birth, we registered mode of delivery, stillbirth, low Apgar score < 7 at 5 min, sex of the child, low birth weight (< 2500 g), the birth weight and length and the placental weight. Small for gestational age (SGA) was defined as a birth weight below − 15% (10th percentile) of the expected for gestational age and sex [23].

### T2*-weighted magnetic resonance imaging and ultrasound assessment

MRI is regarded safe during pregnancy, and to date, no human or animal studies have shown any association between MRI and adverse foetal outcomes [24, 25].

Placental T2* measurements were planned at GW24 and GW32; however, due to logistic challenges, the scans were not performed exactly on the particular gestational time points. Still, the median GW at MRI did not differ between groups at the two examinations. Differing times are accounted for in the “Results” section.

All T2* procedures were conducted at the Department of Radiology, Aarhus University Hospital, Denmark, as procedures dedicated to the present study. All T2* measurements were performed using a General Electric (GE) 1.5 T platform (GE Optima MR450w, Chicago, IL, USA) and multi-echo gradient recalled echo sequence with the following parameters: repetition time = 70.9 ms; 16 echoes ranging from 3.0 to 67.5 ms in steps of 4.3 ms; flip angle 30°; field of view 380 × 380 mm and matrix 256 × 160 mm. We obtained five 8-mm central placental slices perpendicular to the axis of the placenta to ensure maximal tissue representation and with a slice gap of approximately 2 cm. Each slice was acquired in a single breath-hold of 12 s.

**Fig. 1:**
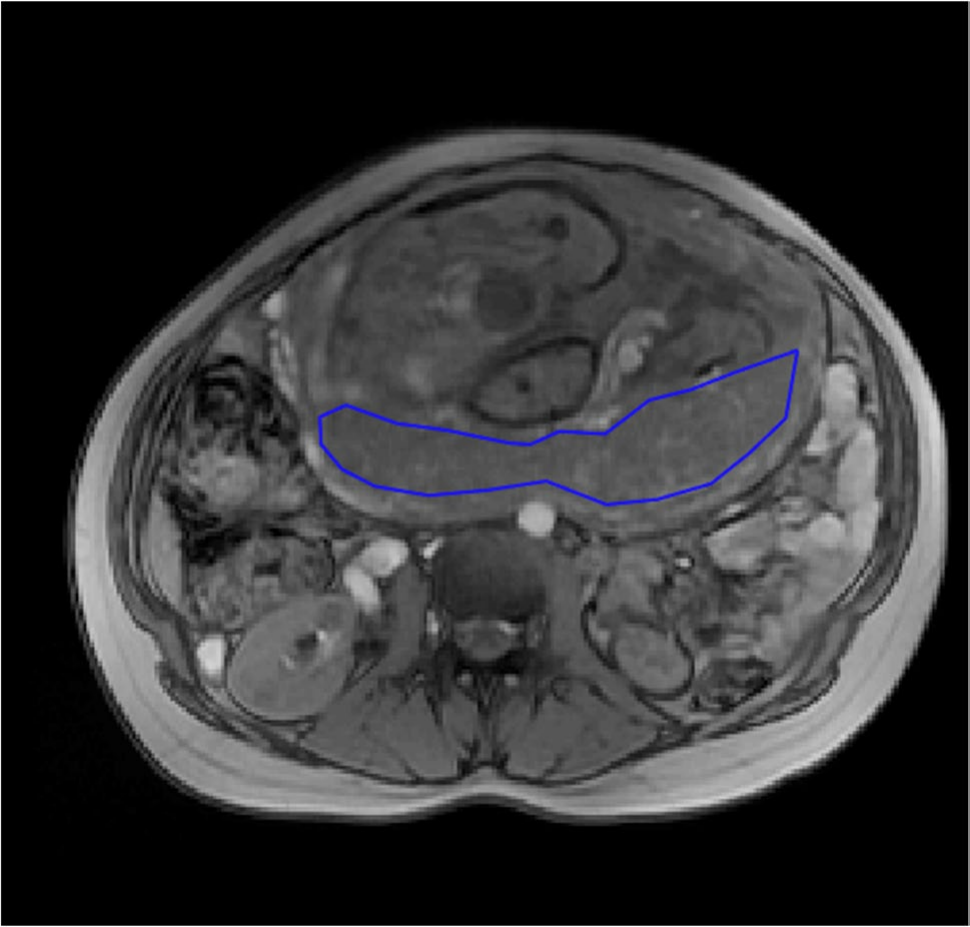
T2*-weighted magnetic resonance image of a transverse section of a pregnant woman, with the outline of the placenta (the region of interest) drawn up

The MRI Dicom data were processed using an in-house developed programme written in MatLab (The MathWorks Inc., Natick, MA, USA). In each placental slice, a region of interest (ROI) covering the entire placenta was drawn, and the placental T2* value was estimated from the mean signal intensities within the placental ROI as a function of echo time using a non-linear least squares fitting algorithm [26]. Subsequently, each slice was assessed for quality, representability and artefacts. In case of placental susceptibility artefacts due to air in adjacent bowels, pulsation from adjacent blood vessels or movement of the foetus, the areas affected were excluded from the ROI. By careful assessment, three representative slices out of the five were selected. The T2* value was reported as the mean of the three acquired placental slices. Were fewer than three slices available, the mean of at least two slices was calculated. All ROIs were drawn and assessed by a single observer, blinded for CRD status and obstetric outcomes TV (see Fig. 1).

All participants had a transabdominal ultrasound examination conducted at the Obstetrical Department of Aarhus University Hospital on the same days as they had their respective MRI scans performed at GW24 and GW32. The ultrasound assessments were procedures performed as part of the present study and not routine examinations. During this examination, estimated foetal weight (EFW) was assessed by Hadlock’s formula [27]. SGA was defined as a foetal weight deviating more than − 15% (10th percentile) from the expected for gestational age at the time of the ultrasound using the reference curve by Marsal et al. [23].

### Statistical analyses

Characteristics of participating women and data on their birth outcomes were tabulated in contingency tables, stratified by group. All continuous covariates were presented as medians with interquartile range (IQR), as only birth weight followed a Gaussian distribution.

Groups of unpaired data normally distributed were compared using simple *t*-tests if equal variance and otherwise using Welch’s correction to obtain *p*-values. When comparing groups of unpaired data of non-Gaussian distribution, non-parametric Mann–Whitney *U* tests or Brunner-Munzel tests were applied depending on the presence of tied values. For comparison of nominal variables, the chi-square test, Fisher’s exact or Wilcoxon rank test was applied depending on sample sizes and distribution of data.

When assessing the effect of disease activity and medical treatment on the T2*P-MRI, the diseased group was stratified accordingly.

Birth weight deviation from expected for gestational age was calculated using the reference by Marsal et al. [23].

We performed separate univariate linear regression analyses to investigate the relationship between T2* values at gestational weeks 24 and 32, respectively, and each of the following variables: having CRD, deviation from expected birth weight, maternal BMI, parity, smoking during pregnancy, biological treatment and disease activity. No multivariate regression analyses were performed as the sample size was too small and any attempted models were overfitted. Spearman’s correlation coefficients were also employed to assess the relationship between the T2* value at GW24 and GW32, respectively, and the deviation from expected birth weight.

The statistics and analyses were made using R for statistical computing (RStudio Team (2020). RStudio: Integrated Development for R. RStudio, PBC, Boston, MA, URL http://www.rstudio.com/). Statistical significance was set at *p* < 0.05.

All figures were created using biorender.com.

## Results

Between the years of 2019 and 2021, we included 10 women with CRD and 18 healthy controls.

Disease activity during pregnancy was present in 3 of the 10 (30%) women with CRD. In the CRD group, 7 out of 10 women (70%) were treated with biologics during pregnancy.

Participant characteristics are shown in Table 1.

**Table 1:** Characteristics of 28 pregnant women stratified according to disease

Number of participants	Controls	CRD
	18	10
*Maternal characteristics*		
Maternal age, median (IQR)	28.5 (26.0; 32.8)	30.7 (28.7; 33.9)
Maternal BMI, median (IQR)	22.68 (20.42–24.64)	23.16 (21.22–25.14)
*Disease type*		
Rheumatoid arthritis, *n* (%)	NA	5 (50.0)
Ankylosing spondylitis, *n* (%)	NA	4 (40.0)
Psoriatic arthritis, *n* (%)	NA	1 (10.0)
*Parity*		
Primiparous, *n* (%)	13 (72.0)	5 (50.0)
Multiparous, *n* (%)	5 (27.8)	5 (50.0)
Smoking during pregnancy, *n* (%)	1 (5.6)	0 (0.0)
Smoking within 6 months of conception, *n* (%)	5 (27.8)	2 (20.0)
*Conception*		
Natural	17 (94.4)	8 (80.0)
Assisted reproduction	1 (5.6)	2 (20.0)
*Antenatal medical treatment*		
Biologics	NA	7 (70.0)
Infliximab	NA	2 (20.0)
Etanercept	NA	3 (30.0)
Certolizumab	NA	2 (20.0)
Local corticosteroid	NA	1 (10.0)
Systemic corticosteroids	NA	3 (30.0)
Sulfasalazine	NA	2 (20.0)
No medical treatment during pregnancy	18 (100.0)	2 (20.0)
*Disease activity anytime during pregnancy*	NA	3 (30.0)
1*st* trimester	NA	1 (10.0)
2*nd* trimester	NA	3 (30.0)
3*rd* trimester	NA	1 (10.0)
*Pregnancy complications*		
Preeclampsia	0 (0.0)	1 (10.0)*
Maternal infection requiring hospital admission	1 (5.6)***	1 (10.0)**
Hyperemesis	1 (5.6)	0 (0.0)

Neither of the above-mentioned women were ultrasound-assessed as carrying SGA foetuses, and only the healthy control who was admitted with gastroenteritis in GW30 gave birth to an SGA neonate—her T2* value was however already in the lower percentile of the healthy controls in GW24 before her infection

*Abbreviation: CRD* chronic rheumatic disease, *IQR* interquartile range

^*^The woman in question was diagnosed with mild preeclampsia in GW41 and thus had labour induction performed

^**^The woman in question had a laparoscopic appendectomy due to appendicitis in GW19, no sequelae or complications

^***^The woman in question was admitted with gastroenteritis in GW30 and was treated with intravenous fluids

### Intra-uterine ultrasound assessments and birth outcomes

Ultrasound EFW was reduced in the CRD group at both GW24 and GW32 when compared to the controls, though not statistically significant. No healthy controls were assessed to be SGA by ultrasound at GW24 or GW32. Two women with CRD were assessed to carry SGA foetuses, one with an EFW of − 15.6 in GW24 and the other with an EFW of − 17.7 in GW32—but only one of them gave birth to an SGA neonate, and the associated intrauterine assessment of being SGA was that of GW32, not GW24. Birth weight was lower in the CRD group, however not statistically significant. Gestational age at birth in the two groups was similar.

The placental weight was also reduced in the CRD group compared to controls, though not with statistical significance. Details are shown in Table 2.

**Table 2:** Birth outcomes stratified according to group

Number of participants	Controls	CRD	*p*-value
	18	10	-
Gestational age at birth (weeks), median (IQR)	40.5 (39.2–41.7)	41.0 (41.0–41.7)	0.3
Birth weight (grammes), median (IQR)	3490 (3243–3836)	3327 (3105–3640)	0.3
Length (centimetres), median (IQR)	52 (50–53)	51 (50–51)	0.2
Placental weight (grammes), median [IQR]	632 (588–698)	555 (525–627)	0.2
Preterm, < 37 gestational weeks, *n* (%)	1 (5.6)	0 (0.0)	1
Small for gestational age, *n* (%)	1 (5.6)	3 (30.0)	0.2
Low birth weight, < 2500 g, *n* (%)	1 (5.6)	0 (0.0)	1
Stillbirth, *n* (%)	0 (0.0)	0 (0.0)	1
Induction of labour, *n* (%)	4 (22.2)	4 (40.0)	0.6
Sectio, *n* (%)	5 (27.8)	2 (20.0)	1
Apgar score at 5 min, < 7, *n* (%)	0 (0.0)	0 (0.0)	1
Sex, *n* (%)			
Girl	11 (61.1)	6 (60.0)	1
Boy	7 (38.9)	4 (40.0)	1
EFW deviation GW24 (%), median (IQR)	− 5.1 (− 7.0; − 0.1)	− 8.3 (− 12.3; − 2.4)	0.3
EFW deviation GW32 (%), median (IQR)	− 5.0 (− 9.9; − 2.7)	− 9.8 (− 12.6; − 5.5)	0.3

*Abbreviation: CRD* chronic rheumatic disease, *IQR* interquartile range

### T2* values

**Fig. 2:**
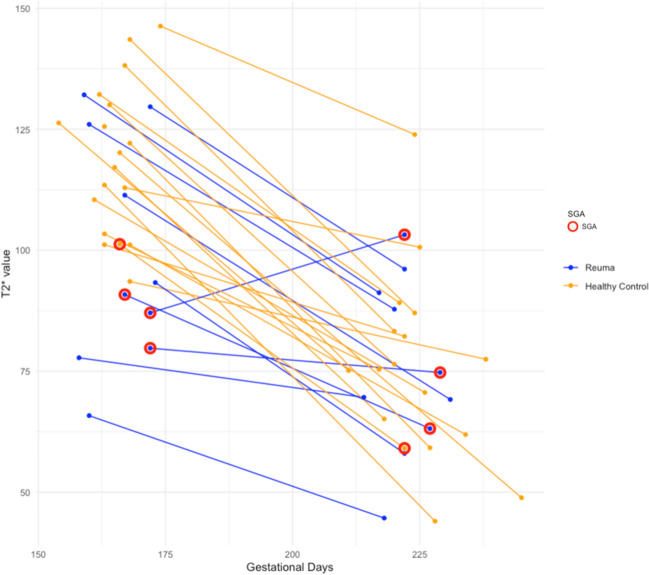
T2* values at gestational week 24 and gestational week 32 in controls (orange) and women with chronic rheumatic disease (blue). Red circles indicate pregnancies that result in children that are small for gestational age (SGA)

**Fig. 3:**
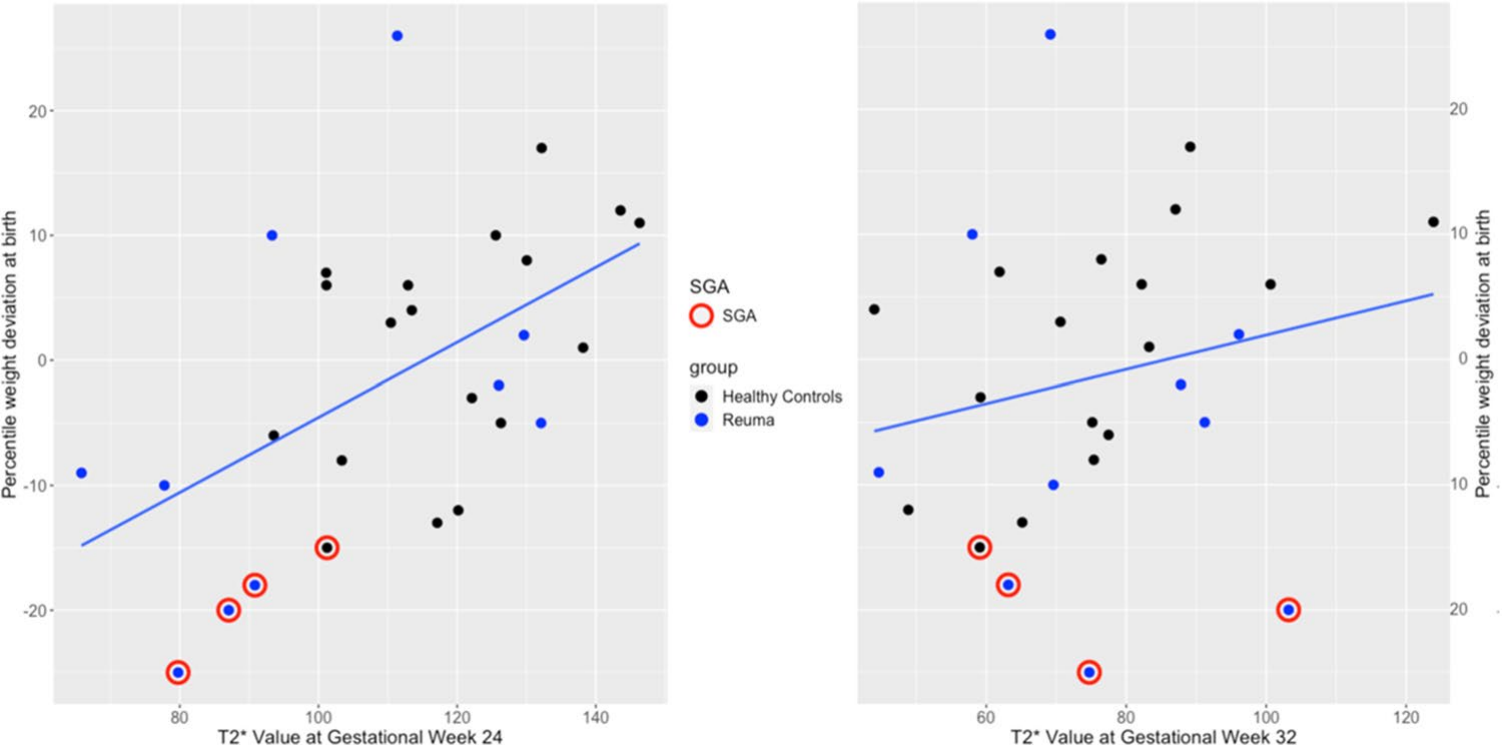
Correlation between ultrasound-assessed deviation from expected birthweight and T2*-weighted MRI at gestational week 24 (*r* = 0.52, *p* = 0.004) and 32 (*r* = 0.05, *p* = 0.29), respectively. Black = healthy controls; blue = chronic rheumatic disease; red circle = small for gestational age

T2* values at GW24 and GW32 are shown in Table 3 and Figs. 2 and 3.

**Table 3:** T2* values and ultrasound-assessed growth restriction at GW24 and GW32 stratified according to group

Number of participants	Controls	CRD	*p*-value
	*N* = 18	*N* = 10	-
*Gestational age 1st MRI (days), median (IQR)*	166 (163–168)	167 (160–172)	0.7
*Gestational age 2nd MRI (days), median (IQR)*	224 (220–227)	222 (219–226)	0.3
T2* value GW24 (ms), median (IQR)	118.6 (105.1–129.1)	92.1 (81.6; 122.4)	0.03
T2* value GW32 (ms), median (IQR)	75.4 (61.9–83.3)	72.1 (64.7–90.4)	0.9

*Abbreviation: GW* gestational week, *CRD* chronic rheumatic disease, *IQR* interquartile range, *MRI* magnetic resonance imaging

Gestational age at the time of each MRI did not differ significantly between the two groups.

T2* values at GW24 were significantly lower in women with CRD than in the healthy controls (median T2*(IQR) 92.1 ms (81.6; 122.4) versus 118.6 ms (105.1; 129.1), *p* = 0.03). At GW32, T2*P-MRI was similar between the groups (median T2*(IQR) 72.1 ms (64.7–90.4) versus 75.4 ms (64.7–90.4), *p* = 0.9).

A linear correlation was demonstrated between the T2*P-MRI at GW24 and birth weight deviation (*r* = 0.52, *p* = 0.004). There was a statistically significant association between T2*P-MRI at GW24 and birth weight deviation (Fig. 3). The T2* value at GW24 was a significant predictor of SGA at birth (RR of 0.93 (0.84; 0.99), *p* = 0.04). No linear relationship was demonstrated between the T2*P-MRI at GW32 (*r* = 0.05, *p* = 0.29), and the association was not statistically significant at GW32 when performing regression analysis.

One participant had reversed T2* values at GW24 and GW32, i.e. a higher T2* value at GW32 than at GW24 (Fig. 2). It was hypothesised that the scans may have been analysed erroneously, but in spite of numeral attempts of redrawing the ROIs, the results were sustained and were thus considered an example of biological variation that one ought not to exclude. Also, when excluding said participant on an experimental basis, the difference between the GW24 T2* values of the healthy controls and women with CRD without biological treatment remained of statistical significance. The participant in question was thus kept in all calculations.

#### Disease activity and biological treatment

T2* values stratified by disease activity and biological treatment are shown in Supplementary Table 1–2 and Supplementary Fig. 1. When stratified according to disease activity in the CRD group, we found a non-significant reduction in the T2* value in CRD women with disease activity compared to women in remission in GW24 (median (IQR) 93.3 ms (85.3; 120.5) versus 118.6 ms (105.1; 129.1), *p* = 0.07) (Supplementary Table 1).

When stratified by biological treatment in the CRD group, both treated and untreated women with CRD had lower median T2* values than the controls; however, a statistically significant reduction was only found in CRD women not treated with biologics compared to controls in GW24 (untreated CRD, median T2* 93.3 ms IQR (86.5; 102.3) and controls, median T2* 118.6 ms IQR (105.1; 129.1), *p* = 0.03) (Supplementary Table 2).

## Discussion

In the present study, we found decreased placental T2* MRI values in women with CRD compared to healthy controls. As FGR is closely tied to placental tissue hypoxia and, consequently, reduced T2* values, our results indicate decreased placental oxygenation in the CRD group during GW24 [28, 29].

When factoring in biological treatment and disease activity, the differences in T2* values persisted. These findings suggest that the disease in itself may influence the T2* values, and the decreased T2* values revealed in our study may thus offer an explanatory basis for the inclination towards FGR in women with CRD. Interestingly, the T2* values at GW32 were comparable in the groups, and the normal physiological decrease of T2* value seen in healthy pregnancies is thus smaller in placentas of women with CRD due to a lower starting point. Nevertheless, the birth weight and the placental weight of the children of the women with CRD were also lower compared to the healthy controls, and though the difference was not of statistical significance, it may provide a suspicion of placental dysfunction as represented by a decreased T2* value in the CRD group.

As the particular subject has never been investigated before, comparisons with previous studies are not possible, but our results were nevertheless within the range of other larger studies, thus validating the method [9, 13]. The T2* values of ultrasound-assessed SGA-pregnancies were by way of example investigated by Hansen et al., who also found the T2*P-MRI to be predictive of SGA neonates, and this in a cohort of normal uterine artery Doppler flows, thereby underlining the superior sensitivity of the T2*P-MRI method in demasking risk pregnancies otherwise not found by ultrasound assessment [9].

In the current study, our findings also established a significant link between the T2* values of the placenta and birth weight, thereby reinforcing the reliability of T2*-weighted placental MRI as an indicator of placental function [9, 13]. Furthermore, we demonstrated the capability of identifying pregnancies at risk by revealing a substantial association between the occurrence of small for gestational age (SGA) and low T2* values. Particularly noteworthy is the fact that this significant association was evident as early as in the 24th week of gestation, enabling clinical assessment and intervention during the second trimester rather than waiting until the routine ultrasound EFW in the 3rd trimester. Previous studies have already highlighted the superior diagnostic potential of T2*-based MRI assessments compared to ultrasound [12]. Interestingly and perhaps as a substantiation of this claim, the children identified as at risk of FGR using T2* placental MRI during GW24 did not exhibit ultrasound signs of SGA at this time point.

The present study has several strengths. Previous studies have established that an average calculated from several MRI slices is more representative of the true T2* value, as the intra-individual variation of T2* value within a small area of the placenta is substantial [14]. Our study calculated an average based on three slices and hence presents more solid data. Our cohort is also very well-described in pregnancy-related outcomes as well as disease activity and medications administered in the group of diseased women.

Limitations must also be mentioned. The sample size of the study is small; however, even this small sample size provided statistically significant results. Future studies including greater numbers of women with CRD are in demand to investigate the matter in depth. Furthermore, since disease activity is a potential confounder that affects both choices of medical treatment as well as foetal growth restriction, it would have been of interest to assess the impact of this factor beyond the measures of stratification that we performed. One of the women who gave birth to an SGA neonate experienced disease activity during both her second and third trimester, and we cannot with the present study distinguish if the cause of the foetal growth restriction was placental dysfunction, disease activity or an interplay of the two.

The generalizability must as a consequence be said to be limited, and the overall results serve to raise curiosity and questions rather than providing firm answers.

In conclusion, women with chronic rheumatic disease have significantly lower T2* values of their placentas in GW24 compared with healthy controls, suggesting decreased oxygenation and reduced placental function. T2*-weighted placental MRI identified foetuses at risk of SGA as early as GW24, thereby perhaps offering the potential of additional decision aid in the assessment of pregnant women with CRD.

### Supplementary Information

Below is the link to the electronic supplementary material.

Supplementary file1 (DOCX 120 KB)

## Data Availability

Deidentified individual participant data can be made available upon request to the corresponding author with investigator support after approval of a new study proposal, signed agreement from each participant and with a signed data access agreement with the participating sites.
