# Supplementary material for: Reduced T2*-weighted placental MRI predicts foetal growth restriction in women with chronic rheumatic disease—a Danish explorative study
Source: Clin Rheumatol. 2024 Apr 26;43(6):1989–97. doi: 10.1007/s10067-024-06889-5 (PMC11111562; doi:10.1007/s10067-024-06889-5)
Supplement: Supplementary file 1 — Supplementary file1 (DOCX 120 KB) [file 10067_2024_6889_MOESM1_ESM.docx]

| **Supplementary Table 1**. T2* values at GW24 and GW32 stratified according to disease activity. Comparisons are made between  controls and the respective groups. | | | | | |
| --- | --- | --- | --- | --- | --- |
|  | **Controls** | **Disease Activity** | **p-value** | **Remission** | **p-value** |
| **Number of participants** | N = 18 | N = 3 | - | N = 7 | - |
| **Gestational Day of 1^st^ MRI, median [IQR]** | 166 [163-168] | 160 [159-166] | 0.7 | 167 [164-172] | 0.4 |
| **Gestational Day of 2^nd^ MRI, median [IQR]** | 224 [220-227] | 220 [217-221] | 0.1 | 222 [220-228] | 0.7 |
| **T2* Value GW24, median [IQR]** | 118.6 [105.1-129.1] | 87.1 [82.4-106.5] | 0.2 | 93.3 [85.3-120.5] | 0.07 |
| **T2* Value GW32, median [IQR]** | 75.4 [61.9-83.3] | 87.8 [78.7-95.5] | 0.3 | 69.2 [60.6-83.0] | 0.6 |

| **Supplementary Table 2.** T2* values at GW24 and GW32 stratified according to biological treatment. Comparisons are made between controls and the respective groups. | | | | | |
| --- | --- | --- | --- | --- | --- |
|  | **Controls** | **Biological Treatment** | **p-value** | **No Biological Treatment** | **p-value** |
| **Number of participants** | N = 18 | N = 7 | - | N = 3 | - |
| **Gestational Day of 1^st^ MRI, median [IQR]** | 166 [163-168] | 160 [160-170] | 0.7 | 172 [170-173] | 0.06 |
| **Gestational Day of 2^nd^ MRI, median [IQR]** | 224 [220-227] | 220 [218-222] | 0.07 | 229 [226-230] | 0.5 |
| **T2* Value GW24, median [IQR]** | 118.6 [105.1-129.1] | 90.8 [82.4-127.8] | 0.2 | 93.3 [86.5-102.3] | 0.03 |
| **T2* Value GW32, median [IQR]** | 75.4 [61.9-83.3] | 90.4 [87.8-93.7] | 0.5 | 69.2 [63.6-71.9] | 0.3 |

**
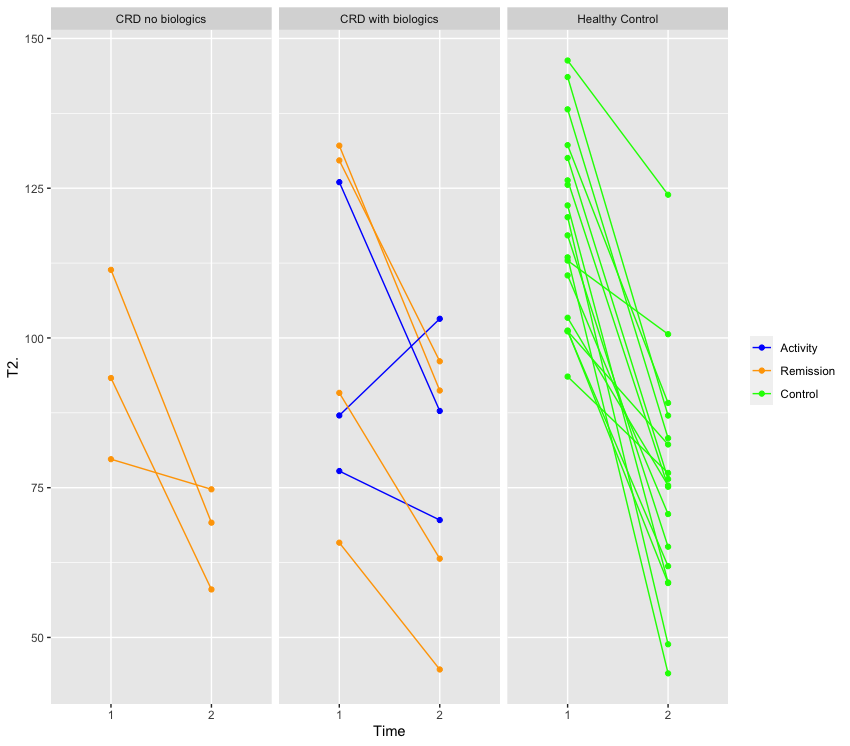
**

***Supplementary Figure 1:*** *T2*-values at GW24 and GW32 stratified by both biological treatment and disease activity during pregnancy.*
